# Acute Water Intoxication Leading to Dilutional Hyponatremia in a Patient With Urinary Tract Infection: A Case Report

**DOI:** 10.7759/cureus.106185

**Published:** 2026-03-31

**Authors:** Sanju Sharma, Preeti Kumari, Faseehullah Alam, Priyanka Dev, Neha Rawat

**Affiliations:** 1 Anaesthesiology &amp; Critical Care, North Eastern Indira Gandhi Regional Institute of Health and Medical Sciences, Shillong, IND; 2 Anaesthesiology &amp; Critical Care, Indira Gandhi Institute of Medical Sciences, Patna, IND; 3 Anaesthesiology, Netaji Subhas Medical College &amp; Hospital (NSMCH), Patna, IND; 4 Anaesthesiology &amp; Critical Care, North Eastern Indira Gandhi Regional Institute of Health & Medical Sciences, Shillong, IND

**Keywords:** acute water intoxication, cerebral edema, dilutional hyponatremia, hypertonic saline, osmotic demyelination syndrome, psychogenic polydipsia, seizure, uroflowmetry

## Abstract

Acute water intoxication is an uncommon but potentially fatal condition characterized by excessive water consumption over a brief period. It results in dilutional hyponatremia. The condition is frequently associated with psychiatric disorders, endurance athletics, or iatrogenic causes. Early recognition and prompt treatment are crucial for preventing serious complications and ensuring favorable outcomes.

A 38-year-old male patient presented to the emergency department exhibiting sudden loss of consciousness followed by altered mental status, confusion, and an episode of seizure. The history revealed ingestion of approximately 8 liters of water within a three-hour period while waiting for uroflowmetry testing. Initial laboratory tests indicated severe hyponatremia with serum sodium of 120 mEq/L. He was diagnosed with acute water intoxication with dilutional hyponatremia. Management involved fluid restriction, electrolyte monitoring, and gradual correction of sodium levels utilizing 3% hypertonic saline. The patient's neurological status improved progressively, and he was discharged after five days.

This case highlights the significance of identifying acute water intoxication as a potential etiology of dilutional hyponatremia, particularly in patients presenting with neurological symptoms and a history of excessive fluid intake. Expeditious diagnosis and appropriate management are essential to prevent severe complications such as cerebral edema and permanent neurological damage.

## Introduction

Acute water intoxication is an uncommon but potentially fatal condition characterized by excessive water consumption over a brief period. It can occur due to numerous etiologies but is challenging to diagnose, as it presents with vague symptoms of altered mental status, disorientation, confusion, nausea, and vomiting, which may resemble psychosis or other neurological conditions [1]. It is more commonly associated with psychiatric illnesses such as psychogenic polydipsia, endurance sports (e.g., marathon running), and ecstasy (3,4-methylenedioxy​methamphetamine (MDMA)) abuse [2, 3]. Although not common, excessive iatrogenic fluid administration during medical procedures can also inadvertently cause water intoxication.

Hyponatremia refers to a reduction in serum sodium concentration below 135 mEq/L and represents the most commonly encountered electrolyte imbalance in hospitalized patients [4, 5]. The kidneys of a healthy adult can eliminate approximately 0.8-1.0 L of free water per hour, depending on solute load and antidiuretic hormone activity, equating to a maximum daily excretion of approximately 18 liters [6]. When this capacity is overwhelmed, as in excessive water intake over a short period, acute hyponatremia can develop rapidly.

The pathophysiology of acute water intoxication involves a rapid influx of water into the body, which overwhelms the kidneys' ability to excrete excess fluid. This leads to a dilution of electrolytes, particularly sodium, in the extracellular fluid, causing hyponatremia. Among the various etiologies, acute water intoxication is a relatively rare but important cause. As the serum sodium concentration decreases, water moves into cells via osmosis, causing cellular swelling. This phenomenon is particularly dangerous in the brain, where expansion is constrained by the rigid cranial vault, potentially leading to cerebral edema and elevated intracranial pressure [7, 8]. Acute hyponatremia is considered a neurological emergency when serum sodium falls below 125 mEq/L, carrying a significant risk of seizures, herniation, and death if untreated [9]. 

Symptoms of acute water intoxication can range from mild to severe and may include headache, nausea, vomiting, confusion, disorientation, seizures, and, in extreme cases, coma or death. The severity of symptoms often correlates with the degree of hyponatremia and the rapidity of its onset. 

Here we are reporting an unusual presentation of acute water intoxication in a middle-aged man who ingested excessive water in anticipation of uroflowmetry testing for urinary tract infection (UTI) symptoms. This case has important implications for clinical practice, particularly regarding patient counseling prior to diagnostic procedures. The case emphasizes the importance of considering water intoxication in patients presenting with sudden neurological deterioration and a suggestive history. Early recognition and prompt treatment are crucial for preventing serious complications and ensuring favorable outcomes. It is important to distinguish acute excessive water intake, as seen in this case, from primary polydipsia, which typically represents a chronic behavioral or psychiatric condition.

## Case presentation

A 38-year-old male patient with no significant past medical history presented to the emergency department with sudden loss of consciousness. This was followed by altered mental status, confusion, and a witnessed generalized tonic-clonic seizure occurring whilst he was awaiting uroflowmetry testing for lower urinary tract symptoms attributed to a UTI. His companion reported that he had been instructed to maintain a full bladder for the procedure and had consequently ingested approximately 8 liters of water over a three-hour period immediately preceding the onset of symptoms. There was no antecedent history of psychiatric illness, substance abuse, malignancy, recent head trauma, fever, or recent travel. He was not taking any regular medications.

On presentation, the patient was confused and unable to give a coherent history. Vital signs were as follows: temperature 36.8°C, heart rate 92 beats per minute, blood pressure 130/85 mmHg, respiratory rate 22 breaths per minute, and oxygen saturation 98% on room air. The Glasgow Coma Scale (GCS) score was 12/15 (E3V4M5). Neurological examination revealed disorientation to time and place with sluggish bilateral pupillary responses, but no focal neurological deficits were identified. There were no signs of meningism. The remainder of the systemic examination was unremarkable. The clinical picture was consistent with an acute encephalopathic process.

Urgent laboratory investigations revealed severe hyponatremia with a serum sodium concentration of 120 mEq/L alongside a correspondingly low serum osmolality of 252 mOsm/kg. Urinary sodium was 47 mEq/L with a urine osmolality of 240 mOsm/kg, suggesting relatively dilute urine; however, this finding alone does not reliably distinguish primary polydipsia from syndrome of inappropriate antidiuretic hormone secretion (SIADH) and must be interpreted in the context of clinical history and additional investigations. Thyroid-stimulating hormone (TSH) was within normal limits; however, free thyroxine (T4) was not assessed, and therefore, central hypothyroidism cannot be completely excluded. Nonetheless, in the absence of clinical features suggestive of hypothyroidism, it was considered unlikely to have contributed to the presentation. The morning serum cortisol levels were within normal limits, effectively ruling out adrenal insufficiency as a contributing etiology. Renal function, blood glucose, and hematological parameters were unremarkable. A non-contrast computed tomography scan of the brain demonstrated no evidence of cerebral edema, intracranial hemorrhage, or space-occupying lesion. A complete summary of laboratory findings is presented in Table 1.

**Table 1:** Laboratory Investigations at Presentation Na⁺: sodium; TSH: thyroid-stimulating hormone; WBC: white blood cell count

Investigation	Patient Value	Reference Range	Status
Serum sodium (Na ⁺)	120 mEq/L	135–145 mEq/L	↓ Low
Urinary sodium (Na ⁺)	47 mEq/L	> 20 mEq/L	Normal
Urine osmolality	240 mOsm/kg	300–900 mOsm/kg	↓ Low
Serum potassium (K) ⁺)	3.8 mEq/L	3.5–5.0 mEq/L	Normal
Serum osmolality	252 mOsm/kg	280–295 mOsm/kg	↓ Low
Blood urea nitrogen	9 mg/dL	7–20 mg/dL	Normal
Serum creatinine	0.9 mg/dL	0.7–1.2 mg/dL	Normal
TSH	2.1 mIU/L	0.4–4.0 mIU/L	Normal
Morning cortisol	18.4 µg/dL	6–23 µg/dL	Normal
Blood glucose	96 mg/dL	70–100 mg/dL	Normal
Hemoglobin	13.8 g/dL	13.5–17.5 g/dL	Normal
WBC count	9,200 /µL	4,500–11,000/µL /µL	Normal

Based on the clinical history, physical examination, and laboratory findings, a diagnosis of acute water intoxication leading to dilutional hyponatremia was established. The diagnosis was supported by the unequivocal history of massive water ingestion (approximately 8 liters over three hours), the absence of edema or signs of volume overload inconsistent with a hypervolemic state, and the dilute urine profile (urine osmolality < 300 mOsm/kg and urinary sodium > 20 mEq/L), arguing against SIADH. Normal TSH and cortisol levels excluded endocrine causes. The patient had no history of psychiatric illness, malignancy, pulmonary disease, central nervous system disorders, or offending medications. The clinical findings were most consistent with acute water intoxication secondary to excessive fluid ingestion. 

The patient was transferred to the intensive care unit (ICU) for close monitoring and active management. A structured treatment protocol was implemented. Fluid restriction to less than 800-1000 mL per day was enforced immediately to prevent further water overload and to reduce the risk of progression of cerebral edema. Intravenous furosemide was administered as an adjunctive measure to promote free water excretion. Although there was no clear evidence of volume overload, its use was intended to enhance water clearance in the setting of excessive fluid intake. Serum sodium levels were closely monitored to avoid overly rapid correction. A Foley catheter was inserted for accurate monitoring of urine output in the intensive care setting.

Gradual correction of hyponatremia was achieved using 3% hypertonic saline administered via a central venous catheter. The target rate of sodium correction was set at no more than 6-8 mEq/L in the first 24 hours, in strict adherence to established guidelines, to minimize the risk of osmotic demyelination syndrome (ODS) [9, 10]. Serum sodium, potassium, and plasma osmolality were monitored every four to six hours throughout active treatment.

Antiepileptic therapy with phenytoin and levetiracetam was initiated to prevent further seizure activity. By 12 hours from admission, serum sodium had risen to 124 mEq/L, and by 24 hours it had reached 128 mEq/L, a controlled rise of 8 mEq/L, within the safe correction threshold. Neurological symptoms improved gradually, and the patient regained full orientation within 36 hours. Hypertonic saline was subsequently tapered, and oral sodium supplementation was commenced. Fluid restriction was maintained for an additional 48 hours. No rebound hyponatremia, osmotic demyelination, or other neurological sequelae were observed.

The patient was discharged from the ICU on day 5 with a serum sodium of 136 mEq/L and a normal neurological examination. Prior to discharge, he received structured counseling regarding safe hydration practices and the risks associated with excessive fluid intake. Table 2 summarizes the serial sodium correction and corresponding clinical progress.

**Table 2:** . Serial Serum Sodium Correction and Clinical Progress GCS: Glasgow Coma Scale; Na⁺: sodium; NaCl: sodium chloride (hypertonic saline)

Time Point	Hours	Serum Na⁺ (mEq/L)	Clinical Status	Intervention
Admission	0	120	GCS 12, seizure, confusion	3% NaCl initiated
12 hours	12	124	Improving orientation	3% NaCl continued
24 hours	24	128	Alert, improving	Rate reduced
36 hours	36	131	Fully oriented	NaCl tapered; oral salt
Day 5 (Discharge)	120	136	Normal neuro exam	Discharged

## Discussion

Water intoxication is a life-threatening yet entirely preventable condition that can rapidly progress to cerebral edema, seizures, coma, or death. This case is notable in that it represents a non-psychiatric, iatrogenically precipitated instance of acute water intoxication in a healthy adult with no pre-existing renal, endocrine, or psychiatric comorbidities, a relatively uncommon scenario in published literature [11].

Several cases of acute water intoxication resulting in severe hyponatremia have been reported in the literature, most commonly in patients with psychiatric disorders such as psychogenic polydipsia or in endurance athletes participating in prolonged events [2, 3]. However, cases occurring in otherwise healthy individuals due to excessive fluid intake prior to diagnostic procedures are relatively uncommon. Similar reports have described hyponatremia following excessive hydration before medical tests requiring bladder filling. These cases, like the present one, emphasize the importance of careful patient instruction regarding appropriate fluid intake before such procedures.

Excessive ingestion of hypotonic fluids overwhelms the renal capacity for free water clearance, which in healthy adults is approximately 0.8-1.0 L per hour, or up to 18 L per day [6]. When fluid intake exceeds this threshold acutely, plasma osmolality drops rapidly. The brain, enclosed within the rigid cranial vault, is particularly susceptible to osmotic shifts; even modest cellular swelling can generate dangerous elevations in intracranial pressure [7, 8]. Clinical severity depends not only on how low the sodium level falls but also on how quickly the decline occurs. Acute hyponatremia (developing over less than 48 hours) is associated with greater morbidity and mortality compared to chronic hyponatremia of equivalent depth, owing to incomplete cerebral adaptation [9].

In the present case, the patient ingested approximately 8 liters of water over three hours, a rate substantially exceeding renal excretory capacity. This was precipitated by a misunderstanding or inadequate specification of pre-procedure hydration instructions for uroflowmetry. Uroflowmetry requires a full bladder at the time of testing, and patients are routinely advised to increase fluid intake beforehand; however, the specific volume and rate are rarely standardized or explicitly defined in clinical practice. This case, therefore, highlights a systemic vulnerability in patient preparation protocols for urological investigations.

The differential diagnosis of hyponatremia in this patient required systematic exclusion of alternative etiologies. Euvolemic hyponatremia, the relevant category given the clinical presentation, encompasses SIADH, primary (psychogenic) polydipsia, hypothyroidism, and adrenal insufficiency as principal causes [12, 13]. SIADH was effectively excluded by the finding of dilute urine (osmolality 240 mOsm/kg; a value well below 100 mOsm/kg would indicate maximal dilution, while values below serum osmolality exclude antidiuretic hormone excess) together with the absence of relevant precipitants such as malignancy, pulmonary disease, CNS pathology, or causative medications [14]. Thyroid and adrenal function testing were both within normal limits. Primary polydipsia was considered the most appropriate diagnostic label, though its precipitant was procedural rather than psychiatric, a distinction of both clinical and medicolegal significance.

Management of acute severe symptomatic hyponatremia is generally guided by established international consensus guidelines [9, 10, 15]. In patients with neurological compromise, including seizures, altered consciousness, or GCS impairment, as in our patient, 3% hypertonic saline is the treatment of choice. Current European guidelines recommend an initial rapid infusion of 150 mL of 3% sodium chloride (NaCl) over 20 minutes in patients with acute severe neurological symptoms, with reassessment and repeat dosing as required until symptoms stabilize [9]. Thereafter, sodium correction should be limited to 6-8 mEq/L in the first 24 hours and a maximum of 10-12 mEq/L in any 24-hour period to mitigate the risk of ODS, previously termed central pontine myelinolysis [10,16]. Our patient achieved a safe and clinically effective correction trajectory (8 mEq/L at 24 hours), with full neurological recovery by 36 hours and normalization of serum sodium by day 5.

The adjunctive use of furosemide in this case facilitated free water excretion and helped address any early fluid overload without further depleting sodium. This approach is consistent with the management of primary polydipsia-associated hyponatremia, where loop diuretics can assist by promoting dilute urine output and accelerating the excretion of excess free water [13].

The concomitant use of phenytoin and levetiracetam for seizure prophylaxis warrants brief comment. In hyponatremic seizures, correction of the underlying metabolic derangement is the primary therapeutic strategy, and antiepileptic drugs are generally considered adjuncts rather than definitive treatment [9]. Nevertheless, their use in the acute setting is clinically justified to prevent further convulsive activity while sodium correction is ongoing.

From a systems perspective, this case raises important questions about patient safety in procedural preparation. The prescription of hydration prior to uroflowmetry or other bladder function tests lacks formal standardization across institutions. In the absence of explicit volume and rate instructions, patients may default to excessive fluid intake, particularly if they are anxious about failing the procedure or have been given only general guidance to "drink enough to have a full bladder." Standardized written instructions specifying an appropriate fluid volume (e.g., 300-500 mL consumed 30-60 minutes prior to testing) may help prevent similar occurrences. Patients should also be clearly advised to avoid excessive water intake. When increased hydration is required, the use of appropriately balanced electrolyte-containing fluids may be considered rather than large volumes of hypotonic fluids. Clinicians should also exercise heightened vigilance when managing patients with pre-existing conditions associated with impaired free water clearance, such as heart failure, liver cirrhosis, hypothyroidism, or SIADH, as these populations are at substantially elevated risk [12].

This report has certain limitations. As a single case observation, the findings cannot be generalized to all clinical settings. In addition, although the clinical history strongly suggested excessive water intake as the primary precipitating factor, continuous monitoring of fluid intake prior to presentation was not available. Nevertheless, the clear temporal relationship between fluid ingestion and symptom onset supports the diagnosis.

## Conclusions

This case illustrates that acute water intoxication and dilutional hyponatremia are not confined to patients with psychiatric illness or athletic contexts; they may arise from procedural misunderstanding in otherwise healthy individuals. A thorough clinical history, prompt laboratory evaluation, and systematic exclusion of alternative etiologies are essential for timely diagnosis. Gradual sodium correction using 3% hypertonic saline, guided by frequent electrolyte monitoring, constitutes the cornerstone of safe and effective management. The potential for irreversible neurological injury, including osmotic demyelination syndrome, underscores the necessity of adhering strictly to correction rate guidelines.

This case also illustrates that water intoxication caused by procedural misunderstanding is largely preventable with clear patient instructions. Standardized, evidence-based hydration protocols for diagnostic procedures, particularly those requiring a full bladder, should be developed and implemented across clinical settings. Healthcare professionals involved in prescribing pre-procedural preparation instructions must be cognisant of the risks of overhydration and ensure that patients receive clear, volume-specific guidance.
